# Quantitative risk assessment for infectious disease introduction in animal populations: a comprehensive review

**DOI:** 10.3389/fvets.2025.1648695

**Published:** 2025-10-10

**Authors:** Ana Rita Pinheiro Marques, Laura Gonzalez Villeta, Robin Simons, Verity Horigan, Clazien de Vos, Beate Conrady

**Affiliations:** ^1^Section for Animal Health and Welfare, University of Copenhagen, Frederiksberg C, Denmark; ^2^Animal and Plant Health Agency, Addlestone, United Kingdom; ^3^Wageningen Bioveterinary Research, Wageningen University & Research, Lelystad, Netherlands

**Keywords:** animal health, disease risk, quantitative risk assessment, import risk analysis, introduction risk

## Abstract

**Introduction:**

Quantitative risk assessments (QRA) are valuable decision-support tools for estimating disease introduction risks in animal populations.

**Methods:**

This review analyzed peer-reviewed QRA studies published between 2010 and 2024 that focused on risk of introduction, aiming to identify methodological trends and challenges.

**Results:**

From the 1,933 studies screened, only 34 (2%) met inclusion criteria, primarily assessing risk through movements of live animals (*n* = 20), animal products (*n* = 7), both live animals and their products (*n* = 2), or vectors (*n* = 5), with no studies addressing aquatic animals. Most QRAs focused on livestock (n= 11 ruminants, *n* = 6 swine, *n* = 4 poultry, *n* = 2 equids and *n* = 1 ruminants and swine) and diseases like Foot-and-mouth disease, Rabies, Lumpy skin disease, and African swine fever using stochastic approaches, frequently based on scenario tree and WOAH based methodology and supported by expert opinion. Cluster and network analyses revealed distinct methodological groupings and two main collaboration hubs in Europe and the United States.

**Conclusion:**

Key gaps included limited coverage of certain animal species, pathogens, and consequence assessments, with a predominant focus on import risks. Addressing these limitations can strengthen future QRAs as input for animal disease management.

## 1 Introduction

Outbreaks of animal diseases disrupt production, access to international and regional livestock and animal product markets, and livelihoods, and continuously threaten the well-being of farmers. Effective prevention of infectious disease introduction to countries free of disease or without a previous record of disease occurrence requires methodologies and tools that can assess the risk of disease introduction. In this context, risk is defined as the likelihood of a biological agent with the potential to cause an adverse health effect being introduced to an animal population, including pets, borrowing from the definition of import risk analysis from the World Organisation for Animal Health (WOAH) Terrestrial Animal Health Code (1). The WOAH risk assessment framework encourages consistency and transparency across all stakeholders performing risk assessments, and is based on a model that distinguishes between entry, exposure, and consequence assessments (1–3).

Entry assessment evaluates the probability of a disease-causing agent entering a country or region via one or multiple introduction routes, such as trade or movement of animals, animal products, or vectors. Exposure assessment involves identifying the biological pathways through which animals in the importing country or area may be exposed to an introduced infectious agent and providing the probability estimate of the exposure occurring. Consequence assessment involves detailing the potential adverse animal health, environmental, and socio-economic consequences after exposure along with the estimates of the probability of these consequences occurring (1).

Risk assessments are generally carried out using either qualitative, semi-quantitative, or quantitative approaches. Quantitative risk assessments (QRA) utilize techniques to estimate risks numerically (1), while qualitative risk assessments use non-numerical terms to communicate or describe levels of risk (4). Qualitative approaches have proven valuable for generating timely insights and facilitating a broad conceptual understanding of risk. However, they often lack the methodological precision and analytical rigor characteristic of quantitative techniques. Semi-quantitative methods assign numeric values to qualitative risk estimates and are frequently used when data is unavailable or highly uncertain (3). It is noteworthy that, while semi-quantitative and quantitative approaches provide a numeric risk estimate, there is no clear advantage of these over qualitative methods and caution is advised when interpreting these outputs as they may provide a misleading impression of precision and objectivity (3). With the increase in data availability, computational tools, and modeling techniques, quantitative methods are increasingly expected to yield more precise insights and a deeper understanding of the probability and potential impact of specific risks. This is an essential contribution to informed, high-stakes decision-making compared to qualitative and/or semi-quantitative risk assessment (3). However, quantitative approaches require more time and data compared to qualitative and/or semi-quantitative risk assessment (3).

Horigan et al. (4) performed a systematic review regarding qualitative risk assessment in the veterinary field and indicated that several tools have been developed which attempt to provide robust and clear methodologies for qualitative risk assessment, including mathematical reasoning to incorporate uncertainty. While these advancements enhance the objectivity of qualitative assessments, they still depend on subjective judgment and may lack the precision needed for high-stakes decisions. Given the ongoing advancements in risk assessment methodologies and technologies, the integration of quantitative approaches is imperative for enhancing analytical precision and generating robust, data-driven insights. Such methods facilitate detailed numerical analysis and offer a more rigorous foundation for complex decision-making processes. Thus, a comprehensive literature review was conducted to identify and characterize the QRA methods for evaluating the introduction of animal diseases in countries or regions previously considered free of infectious diseases, through live animals, animal products or vectors. Additionally, the review discusses the requirements and challenges associated with conducting QRA related to disease introduction.

## 2 Methods

To identify relevant QRA publications of infectious disease introduction into animal populations (i.e., livestock, companion animals, wildlife, and aquatic animal populations), a comprehensive literature search was conducted with a focus on the risk of disease introduction into countries or areas previously deemed free of infectious disease at the global level.

The search was limited to publications from 2010 onwards, to obtain the most recent references for the topic. The search was performed on September 27th, 2024, using the scientific online databases of Scopus and PubMed. The search strings used for this comprehensive review in both databases are shown in Table 1. To take advantage of the capabilities of the PubMed MeSH terms classification system, MeSH terms were added to the search to identify publications related to infectious disease. The term “health” was used in the Scopus search as opposed to “infectious communicable diseases” used in PubMed to broaden the search results, since the number of publications identified using “infectious” (*n* = 137) was lower than with “health” (*n* = 1,280).

**Table 1:** Terms^1^ used to identify studies related to quantitative risk assessments of infectious disease introduction in animal populations (i.e., livestock, wildlife, and aquatic animal populations).

Scopus	Risk AND assessment* AND animal* (aquatic AND animal*) AND quantitative* AND health AND “Risk assessment”
PubMed	((“risk assessment” OR (“risk” AND “assessment” AND (“animals” OR “animal*” OR “aquatic animal*”) AND “quantitative*” AND (“Communicable Diseases” OR “disease transmission, infectious” OR “communicable diseases, emerging” OR “Disease Notification” OR “Disease Vectors” OR “Disease Outbreaks” OR “Zoonoses” OR “Disease Reservoirs” OR “communicable diseases, imported”

The search terms employed in this study were adapted from those used by Horigan et al. (4) in their analysis of qualitative risk assessment tools.

^1^Due to the large number of studies available, some search terms shown in table were set in quotation marks to ensure that the search engine returned only items containing adjacent combinations of terms.

The articles identified via the search terms in the databases (referred to as primary articles) were collected and reviewed for duplicates with the online software Rayyan (5). The list of the primary articles was screened by title and abstract to identify publications providing examples of QRAs of infectious disease introduction in animal populations. Publications were excluded if the focus was on (i) human health, including quantitative microbial risk assessments and assessments focused on food microbiology and food safety from a public health perspective; or (ii) estimating risk factors or prevalence, as well as potential for spillover or defining at risk populations; (iii) risk management, prioritization of disease or risk ranking methodologies; (iv) non-infectious diseases; and/or (v) for qualitative or semi-quantitative assessments. Zoonotic diseases, where infectious disease is transmissible under natural conditions from vertebrate animals to humans, were considered when the publication focus was on animal health. After the primary exclusion of publications, a second selection round based on full text screening was performed to identify and exclude studies that did not assess the risk of disease introduction into a country or area previously deemed free of disease and considered only disease spread, exposure, and/or consequence, or were considered as reviews of quantitative risk methods or other related publications. After this secondary exclusion step, the eligible publications were identified and classified under the following five risk types: introduction; introduction and exposure; introduction and consequence; introduction, exposure and consequence; and export (i.e., QRA quantified the risk of exporting a pathogen to another country or region, resulting in the risk of introduction of the pathogen in a destination country). In the present study, risk of entry, risk of release, incursion risk, and introduction of disease are used interchangeably. A relevant pathogen in animal health was defined as “a pathogen impacting animal health and that could be imported or transmitted through livestock (including fish) or animal/fish products” (4). Furthermore, *n* = 4 QRA’s that met the inclusion criteria were identified from 6 review-type publications and included in the final number of publications for analysis.

The following information was collected and analyzed from the final included studies (Supplementary Material 1, Supplementary Table 1).

1. QRA type: if QRA was an IRA (i.e., assessing the introduction via import of live animals and/or animal products) or not (in case introduction via a vector import, via export, or other category)
2. Risk type: entry; entry and exposure; entry and consequence; entry, exposure and consequence; and export
3. QRA methodology

  (1)  If QRA considered aggregation of animals at one level, usually the country, or at several levels of hierarchical groupings, such as herds or farms (multilevel)
  (2)  If decision trees were used to illustrate the risk pathway
  (3)  If spatial analysis was performed
  (4)  If QRA was stochastic or deterministic
  (5)  If sensitivity analysis was done to assess the impact of model inputs
  (6)  If the study assessed QRA for a default scenario or for multiple scenarios through variations of inputs and/or input parameters

4. Tools and resources

  (1)  Software used for estimating QRA
  (2)  If WOAH methodology was applied
  (3)  If expert opinion was used for parameterization

5. Geographical and temporal context

  (1)  Country for which QRA was assessed
  (2)  Spatial unit of the QRA assessment which can vary within a country or consider multiple areas
  (3)  Time period for which QRA was assessed

6. Pathway data

  (1)  If vectors were considered for disease introduction
  (2)  If wild or feral hosts were considered at any risk stage
  (3)  If wild animal movement was considered for disease introduction
  (4)  If wind dispersion was considered for disease introduction
  (5)  If legal livestock movements were considered
  (6)  If legal animal product movements were considered
  (7)  If truck movements were considered
  (8)  If flight data (aircraft) were considered
  (9)  If maritime data (ships) were considered
  (10)  If undocumented or illegal movements of live animals and/or animal products were considered

Multiple Correspondence Analysis (MCA) was used to gain deeper insights into the structure and relationships of the collected data from the eligible studies. MCA is a statistical technique used to visualize and analyze the relationships between multiple categorical variables. MCA was performed for the variables in categories (c), (d) and (f), with variables (a) and (b) projected on the MCA plot as supplementary. The active variables were selected to minimize the spread of the variance among many and possibly correlated variable classes. The supplementary variables are projected on the first two dimensions but do not contribute to the construction of the projections on the dimensions. Outliers were identified as the top 5% of publications with the largest distances from the MCA plot origin as well as visually. To better understand the MCA results, hierarchical clustering was performed on the MCA output to identify clusters of similar publications and their characteristics. The MCA was performed using the MCA function from the FactoMineR package in R (6). Subsequently, Hierarchical Clustering on Principal Components (HCPC) was applied using the HCPC function to identify clusters within the data. The suggested cutoff for the number of clusters is automatic and based on inertia gain.

Furthermore, a network analysis was performed to identify research communities based on the country affiliation of the authors involved in the publications in this study. Nodes represent countries, and edges represent the frequency of collaborations across the authors. We calculated degree centrality, which indicates the number of direct connections each node has within the network. Additionally, we assessed the clustering coefficient, a measure that reflects the tendency of nodes to form tightly connected groups or clusters within the network (7) using the Leiden clustering method with a resolution parameter of 1.5. This analysis aims to reveal the geographical distribution of research activity and assesses the intensity of international collaboration. The network analysis was performed in R using the igraph package (8).

Additionally, a Shiny R application (9) was developed to enable users to select, filter, and visualize data, generate frequency plots, and explore Sankey diagrams based on 2 to 4 variables from the eligible studies included in this analysis. This interactive tool facilitates the identification of relevant studies and enhances the understanding of key data patterns and methodological approaches. The app is available at: https://tipton-arpm.shinyapps.io/QRA_data_explorer/.

## 3 Results

**Figure 1:**
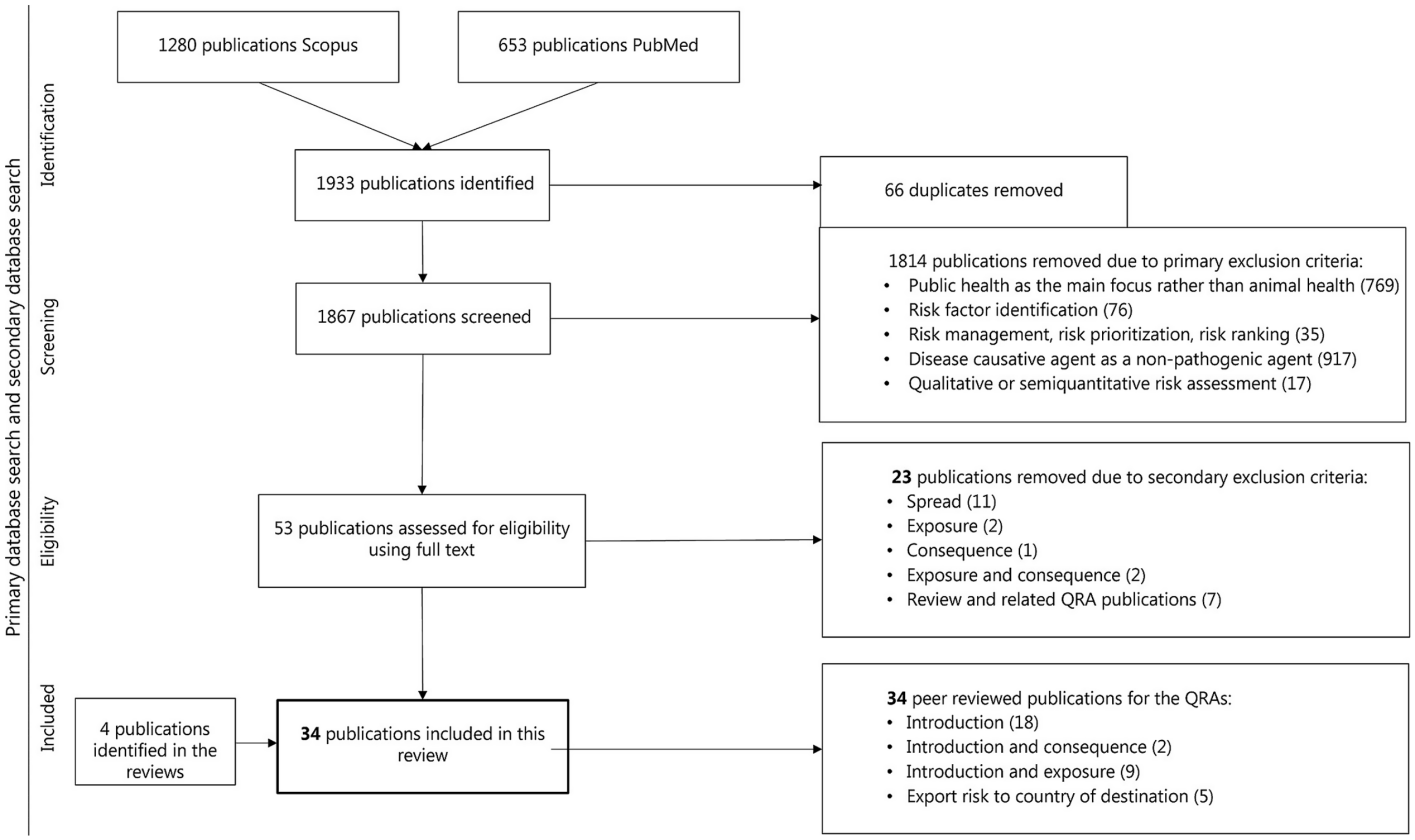
Flow chart of studies incorporated in the comprehensive review of QRA in infectious diseases in animal populations with a focus on the risk of entry or introduction of disease in animal populations through the introduction of live animals or animal products.

A total of 1,933 publications were initially identified. After removing 66 duplicates, 1,867 publications were screened for title and abstract. During the first screening stage, 1,814 publications were excluded based on the primary exclusion criteria. An additional 23 were excluded based on secondary exclusion criteria (Figure 1). Ultimately, 34 publications (2% of the initial records) met the inclusion criteria and were included in the present study, including four eligible review studies.

**Figure 2:**
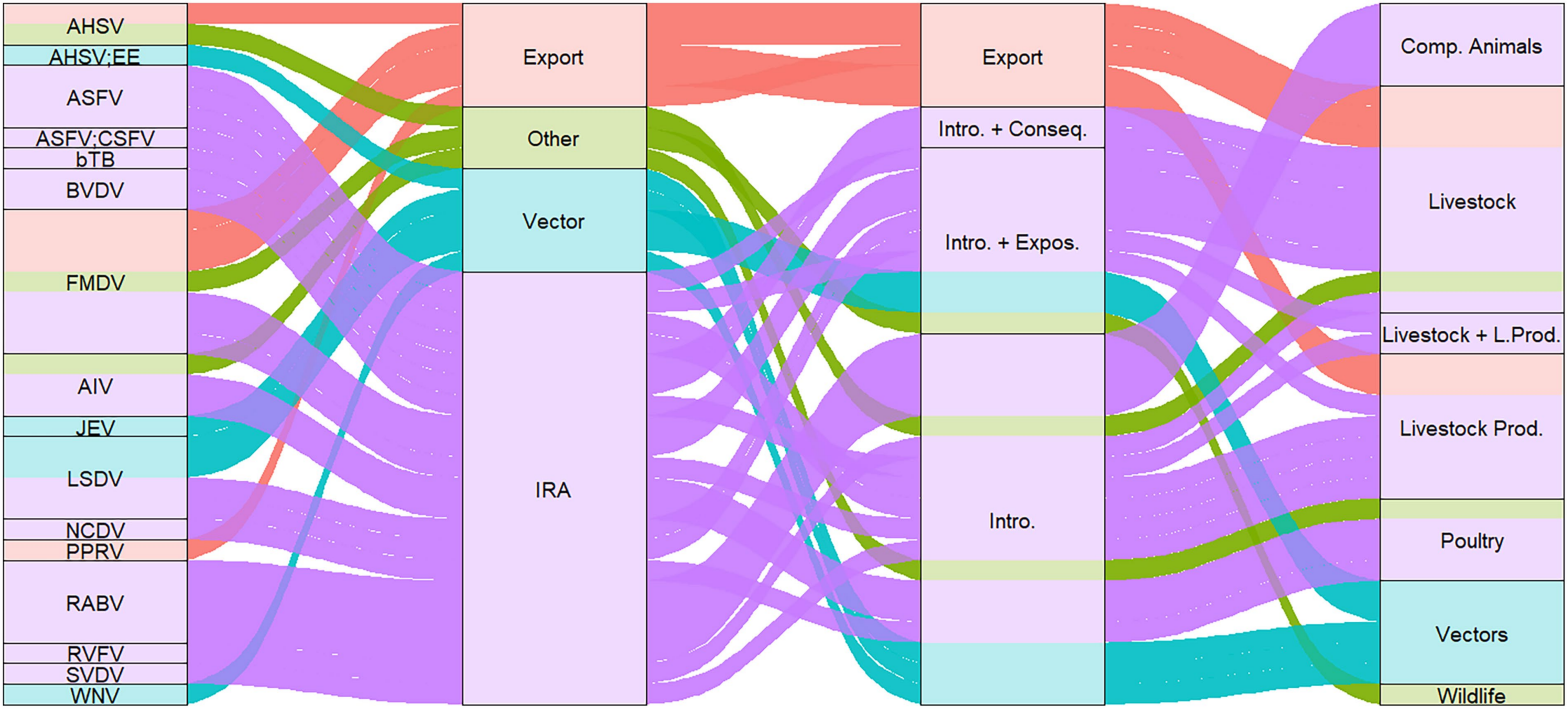
Sankey diagram of publications per (i) pathogen, (ii) QRA purpose, (iii) type of risk assessment, and (iv) risk source types. AHSV, African horse sickness virus; AHSV; EE, African horse sickness virus and Equine encephalitis; ASFV, African swine fever virus; ASFV; CSFV, African swine fever virus and Classical swine fever virus; bTB, Bovine tuberculosis; FMDV, foot and mouth disease virus; AIV, Avian influenza virus; JEV, Japanese encephalitis virus; LSDV, lumpy skin disease virus; NCDV, neonatal calf diarrhea virus; PPRV, Peste des petits ruminants virus; RABV, Rabies virus; RVFV, Rift valley fever virus; SVDV, swine vesicular disease virus; WNV, West Nile virus; Intro. + Conseq., introduction and consequence; Intro. + Expos., introduction and exposure; Intro., Introduction; Comp. Animals, companion animals.

From the 34 studies, 22 studies performed QRA for disease introduction through import of live animals, nine studies through import of animal products and five studies for vector related introduction. Two studies considered both livestock and livestock products in their assessment. In total, 24 studies assessed the introduction risk through live animals and/or livestock (*n* = 11 ruminants, *n* = 6 swine, *n* = 4 poultry, *n* = 2 equids and *n* = 1 ruminants and swine), one study assessed the risk through wildlife, four studies assessed the risk through companion animals, and five studies through vectors (Figure 2). No study was identified that performed QRA for aquatic animals.

In total, 21 studies focused on risk of introduction of diseases through imports, five studies assess the risk through vector migration, and five studies assessed the risk of disease introduction for a destination country through an export analysis (Figure 2). A detailed description of the studies stratified by introduction risk through imports and exports of livestock, animal products, vectors and others can be found in the Supplementary Materials 1.

The QRAs were performed for 16 different pathogens. The majority of QRAs focused on Foot-and-mouth disease virus (FMDV) (*n* = 7), followed by Rabies virus (RABV; *n* = 4), Lumpy skin disease virus (LSDV) (*n* = 4) and African swine fever virus (ASFV) (*n* = 4). Figure 2 shows that all publications assessed the risk of introduction for all pathogens except Peste des petits ruminants virus (PPRV), which was only evaluated for exported animals. Introduction and exposure were considered for LSDV, FMDV, and ASFV, while the introduction risk and associated consequences were assessed for Bovine tuberculosis (bTB) and Bovine viral diarrhea virus (BVDV). The consequences of disease introduction were expressed in one study as economic impact while also analysing the cost-effectiveness of additional testing of imported animals (10). Exposure (*n* = 9) was mainly measured as the probability of contact resulting in transmission. In total, eleven studies assessed the risk for zoonotic diseases [i.e., Highly pathogenic avian influenza virus (HPAIV), Japanese encephalitis virus (JEV), RABV, Rift Valley fever virus (RVFV), Bovine tuberculosis (bTB) and West Nile virus (WNV)].

Assessments were conducted for 17 countries, and the majority of the studies were performed for the United States of America (USA) (*n* = 4) and France (*n* = 4) and two regions [South-East Mediterranean and the European Union (EU)], with most QRAs performed for the EU (*n* = 5). There was no clear trend in the number of publications per year. Thirty-one of the QRAs assessed the risk at an annual level, while six studies considered both monthly and annual assessments. Live animal movement, informed by movement data, was the most frequently considered introduction pathway in the QRA (*n* = 23). Five publications considered introduction of diseases by illegal or undocumented movement of animals through smuggling or non-compliance with animal movement regulations on vaccination status, across borders overlooking inspections, in luggage of air passengers or undocumented movement of people. Flight traffic data (*n* = 5) or introduction through wind dispersal (*n* = 3), truck movement data (*n* = 2) and by maritime traffic (*n* = 2). Numerous studies used expert opinion for parameterization and data inputs for the QRA (*n* = 12). Information on the method for expert elicitation of parameter and probability estimates varied among publications, with some papers reporting from *n* = 2 to *n* = 14 experts, the application of questionnaires in *n* = 5 and probability estimates characterized as Pert distributions in *n* = 6 publications. One study considered expert based risk maps with attribution for geographical layers and weights. Thirty-three studies assessed the impact of input data on the outcome of QRA through a sensitivity analysis. The sensitivity analysis methodology varied with the majority of studies applying Spearman rank order correlations (*n* = 13), followed by regression analysis (*n* = 10) and variation of parameter values (*n* = 10). Both Spearman and variation in parameters methods were applied in one publication, one publication applied spatial sensitivity analysis, and six publications applying further analysis and accounting for stochasticity with @Risk. Eighteen publications assessed risk for multiple scenarios.

Thirteen QRAs referred directly to scenario, event or decision trees to illustrate QRA for introduction, and 11 referred to the WOAH guidelines as the methodology adopted to determine risk. Most of the publications performed stochastic QRA (*n* = 33), and most QRAs ran on Excel software together with @Risk software or were developed using R statistical software (*n* = 23 and *n* = 7, respectively). Three QRA were determined using spatial analysis techniques.

Assumptions played a critical role in risk assessments and modeling, possibly introducing uncertainties or leading to misleading results. A variety of assumptions were identified in the reviewed publications:

1. Transmission, transportation, infectious period and probability of infection (11);
2. Vector survival and dispersal (12);
3. Trade similarities between regions (13);
4. The conditions, region, and time in which the studies were conducted differ from those in the references used for model parameterization but were based on these as proxy values (14);
5. Assumptions about herd-level effects given that multilevel quantitative risk models require compared to one-level models (15);
6. Vector species presence and likelihood of being infected (15);
7. Assumptions about subclinical rates (16);
8. Assumptions about quarantine risk mitigation benefits (16);
9. Assumptions about the prevalence of infection from other countries (17);
10. Assumptions about the biosecurity level of farms at origin (18); and
11. Assumptions about the complexities of disease and disease pathogens (19).

**Figure 3:**
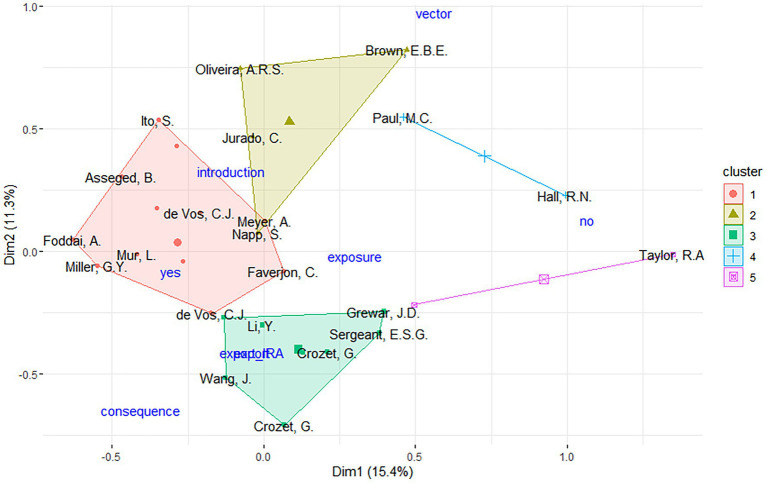
Hierarchical clustering of MCA results, identifying five distinct clusters of publications collaborations. Different publications illustrated with the last name of the first author. Supplementary variables (i.e., QRA type and risk type) are represented in blue color. yes, risk type indicating import risk analysis; no, risk type indicating not import risk analysis; export_IRA, risk type indicating risk as export; vector, risk type indicating IRA related to the introduction of vectors; introduction, QRA type indicating just introduction assessed; exposure, QRA type indicating introduction and exposure assessed; consequence, QRA type indicating introduction and consequence assessed; export, QRA type indicating risk as export.

The MCA analysis identified two outlier publications. After removing the outliers and performing MCA a second time, five distinct clusters of studies were identified, each with unique features (Figure 3):

- Cluster 1 (*n* = 16): This cluster includes all of the publications identified as using multilevel analysis on animal aggregation for risk assessment, as well as 80% of those using WOAH methodology. This cluster also includes *n* = 7 of the publications that use animal product movement data.
- Cluster 2 (*n* = 4): Characterized by publications that included maritime movement data and flight data, respectively 100 and 80% of the studies reviewed.
- Cluster 3 (*n* = 8): Included only assessments using live animal movement data and all of the studies not using the WOAH method.
- Cluster 4 (*n* = 2): All of the studies in this cluster conducted spatial analysis.
- Cluster 5 (*n* = 2): Characterized by studies involving wild animals and wild animal movement data.

One of the key findings is the contrasting nature of publications in Clusters 1 and 3. A large proportion of studies focusing on animal product movement were grouped in Cluster 1, where they commonly applied the WOAH methodology, and used multilevel analysis. In contrast, studies addressing live animal movement and grouped in Cluster 3 were largely unassociated with WOAH-based assessments. For these publications, the supplementary variable projection suggests that export-related QRAs might characterize this cluster even if not significantly.

**Figure 4:**
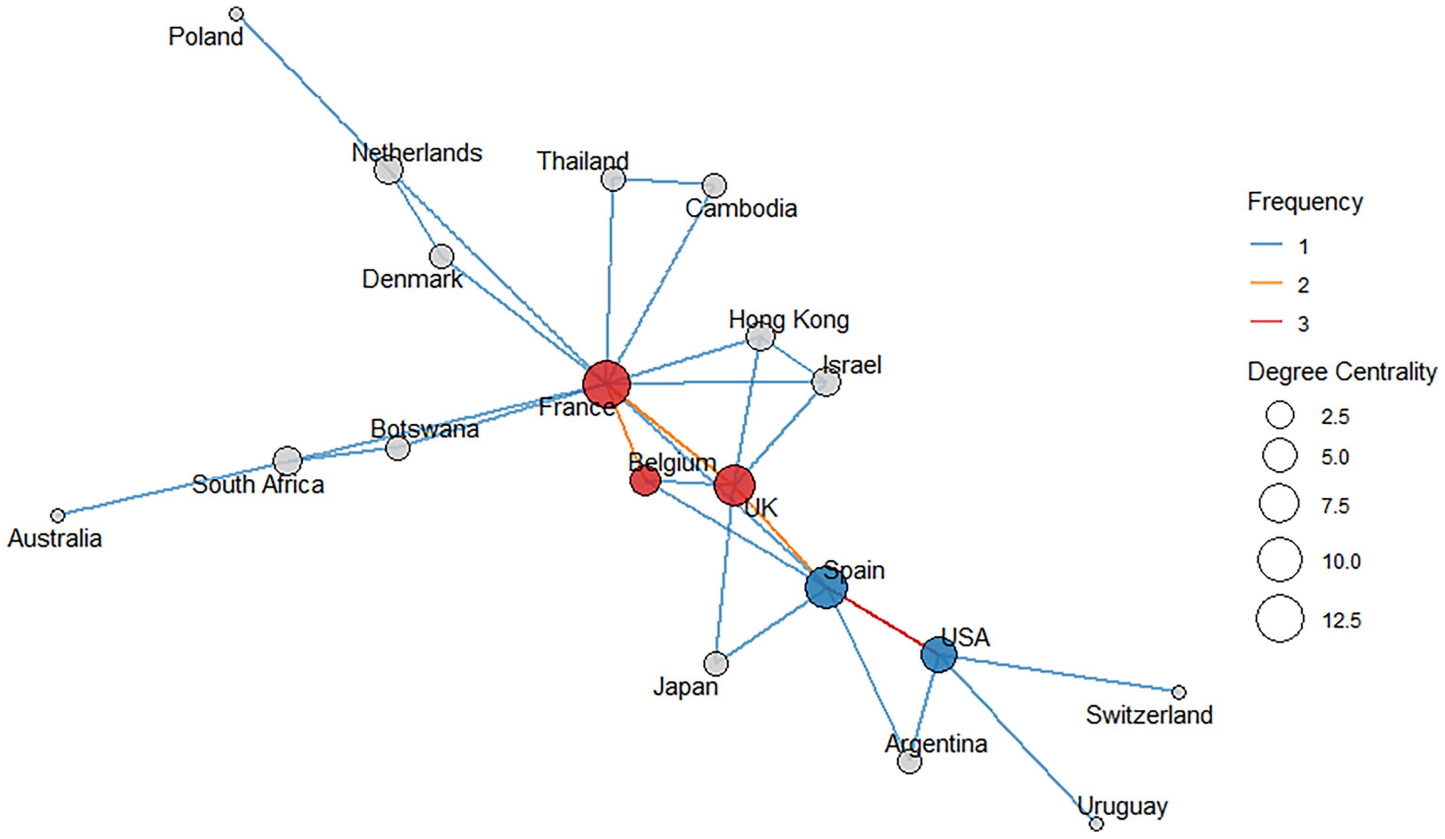
Network of countries represented in the included publications. Each country is shown as a node, and edges represent collaborative links between them. Edge colors indicate the frequency of collaboration, with red lines denoting the highest collaboration frequencies. The size of each node reflects its degree centrality: the higher the degree centrality, the larger the node, indicating more collaborative connections. The blue nodes represent members of Cluster 1, whereas the red nodes denote members of Cluster 2.

The network analysis revealed two main collaboration clusters in QRA (Figure 4):

- Cluster 1 consists of Spain and the USA, with Spain showing a degree centrality of 9 and the USA a degree centrality of 6. These two countries are connected by a single edge with a relatively high interaction frequency of 3.
- Cluster 2 includes the United Kingdom (UK), France, and Belgium, with degree centralities of 8, 13, and 4, respectively. This cluster is connected by three edges, each with a frequency of 2.

Several other countries—Uruguay, Argentina, Denmark, Netherlands, Switzerland, Thailand, Cambodia, Israel, Hong Kong, Australia, South Africa, Japan, Poland, and Botswana—appear in the network with varying levels of centrality, but do not form part of these two main clusters.

## 4 Discussion

Out of the 34 reviewed QRA studies, 20 conducted QRAs for disease introduction via live animals, 7 for animal products, and 2 for both live animals and their products. This indicates a strong emphasis on live animal movement as the primary concern in transboundary disease risk, likely due to the higher perceived likelihood of pathogen transmission through this pathway and to how this is the most easily quantified route. The movement of live animals as the most frequently analyzed pathway for disease introduction also reinforces its central role in animal health biosecurity strategies. Only one study assessed risks associated with wildlife and no QRAs were found for aquatic animals. This highlights a notable gap in risk assessments for wildlife and aquatic species, despite their recognized importance in the global disease ecology landscape. However, the absence of studies specific to aquatic animals may reflect limitations in our definition of pathogen introduction. By applying inclusion criteria primarily oriented toward terrestrial spread and the presence of clearly defined borders, our approach may have excluded insightful quantitative risk assessments in open water environments, where such borders are less clearly applicable. While the current set of publications provides valuable insights, its completeness may be limited by several factors. Keyword selection might have excluded relevant references and database choice could bias coverage toward certain disciplines. Additionally, the exclusion criteria applied also shaped the final reviewed publications.

In total, QRAs addressed 16 different pathogens, with the most frequently studied being FMDV (*n* = 7). The predominance likely reflects prioritization due to FMDV being highly contagious, affecting multiple livestock species, and potential cause severe economic disruption through trade restrictions and production losses. Outbreaks in neighboring or trading partner countries often trigger renewed interest, funding, and risk assessment efforts—as seen with recent FMDV cases in parts of Europe. Additionally, this means that for many known transboundary animal diseases, no QRA has yet been published, underscoring the need for broader pathogen coverage in future risk assessments. Limited knowledge of wild animal populations and their interactions with domestic animals represents another significant gap, particularly in understanding disease transmission dynamics (20). This might explain why no QRA was identified for certain pathways and diseases. QRAs were conducted across 17 countries and two regions (South-East Mediterranean and the EU), with the USA and France having the highest number of country-specific assessments (*n* = 4 each). The EU was the most frequently assessed region (*n* = 5).

In contrast, the network analysis of author-country collaborations revealed two distinct clusters:

- Cluster 1: A collaborative link between Spain and the USA.
- Cluster 2: A grouping of the UK, France, and Belgium.

These clusters highlight geographic patterns of scientific collaboration, which may influence the focus and distribution of QRA efforts globally and emphasize the central roles of specific nations in the research landscape. Thirty-two of the 34 studies lack comprehensive consequence assessments, failing to fully capture the economic, social, and environmental impacts of potential risks. Twenty-six QRA studies focused on import risk assessments, including assessments for exports, indicating a strong emphasis on trade-related risks. This focus may overlook other critical areas such as natural animal movement, human mobility, undocumented or illegal movement, vector introduction, among others. However, it is possible that some QRA studies were not identified due to predefined exclusion criteria, restricted keywords or selected data bases in the present study.

This study identified several assumptions in the identified QRAs. Uncertainty in risk assessments is particularly evident in the use of expert opinion (19, 21) and wide probability limits (22) for parameterization. Uncertainty was frequent when determining infection prevalence during certain periods, sampling sizes, estimation using values from neighboring countries and using data from outbreak reporting systems (13, 15, 17, 18). Authors also identified uncertainty when estimating the number of transported vectors (15) and the probability of disease occurrence in low-risk and very low-risk countries (23), among others. Biases in risk assessments can significantly affect the outcomes and interpretations of studies and were mentioned in several of the reviewed QRAs. As an example, many of the reviewed publications refer to how failing to include information on unregistered or illegal trade can result in selection bias (13, 22–25). It was infrequent for authors to directly identify a bias as such, whereas challenges and limitations were more often reported. Performance bias was identified in the reviews regarding the reliance on expert opinion (21). Detection bias was noted for clinical inspections and the difficulty in detecting clinical signs, particularly when they are not pathognomonic (16, 23). Detection bias was also identified for assumptions about a country’s infection status based on suspected cases in other countries, which introduces further inaccuracies (17).

In general, data-related challenges can have a significant impact on the outcomes of a QRA, creating obstacles in the development of reliable risk estimation models. Several key limitations have been identified in the literature. Issues commonly referred to include lack of or inaccurate data and restricted data availability (13, 15, 23, 25, 26). These limitations highlight the need for improved data collection and management strategies to enhance the robustness of QRA models. A key challenge identified in the development of QRAs is the lack of comprehensive empirical data. For instance, data gaps concerning the probability of vectors entering wind streams have been highlighted as significant sources of uncertainty (12). The reliance on incomplete or fragmented historical records of trade and disease outbreaks has also been recognized as a limiting factor (27). Moreover, inconsistencies in how countries report surveillance, often due to varying surveillance capacities, add to these challenges (18).

While our review identified apparent gaps in species and pathogen coverage within the selected QRAs, it is important to acknowledge that these may not always represent true deficiencies. In some cases, the absence of QRAs may reflect a lack of necessity due to the negligible risk posed by certain species-pathogen combinations. Similarly, the limited application of consequence assessments may not always indicate an oversight. For high-impact diseases, where even a single incursion can have devastating economic and trade consequences, a detailed consequence assessment may be considered redundant. However, in many cases, even simplified consequence assessments could add value in certain scenarios.

In relation to IRA tools and methods, which made up the majority of publications in this review, De Vos et al. (28) recommended several areas for improvement. These included the need for better incorporation of uncertainty and variability, clearer identification of uncertainty sources, and more consistent use of sensitivity analyses. The study also emphasized the importance of enhancing data quality and availability to support more robust and reliable IRA outcomes. Bianchini et al. (29) examined user preferences and challenges associated with conducting risk assessments and utilizing animal health information systems, including the WAHIS, developed by the World Organisation for Animal Health (OIE), EMPRES-i, by the Food and Agriculture Organization (FAO). Their findings suggest that the most valued features in risk assessment tools include the ability to assess introduction and spread pathways, typically integrating multiple data sources. However, combining various risk assessment methodologies to produce comprehensive risk reports remains less common (29). Conducting QRA studies is highly resource-intensive, requiring robust data, specialized scientific expertise, significant time, and adequate funding. These demands often limit their feasibility in low-resource settings. Consequently, this limitation may partly account for the geographic imbalance observed in published QRA assessments, the majority of which originate from higher-income countries. As a result, international collaboration across nations of all income levels is crucial to addressing global disease risks (2). The integration of advanced tools and technologies, such as mathematical modeling (30–32), genome sequencing, socio-economic analyses (33–35), and artificial intelligence, holds promise for enhancing both the precision and effectiveness of QRAs. Such approaches are also likely to encourage broader application, stakeholder participation, and cooperation among sectors of animal production (2).

Further, it is important to mention the need for accurate communication of the results from QRA which can be particularly challenging when given the technical details of the analyses, biases and uncertainties. Probability estimates must include confidence intervals and models must be verified and validated (36). Although quantitative approaches generate numerical risk estimates, they do not inherently provide a clear advantage over qualitative methods. These results should be interpreted with caution, as they may convey a misleading sense of precision and objectivity (3). This highlights the importance of transparency and rigor in QRA, which are essential for making informed decisions. Addressing these and other challenges requires more inclusive data collection and data sharing, as well as improved methods for managing uncertainty and assumptions. Future research should also employ advanced analytical techniques and encourage international collaboration to enhance QRA quality and impact.

The presented study identified knowledge gaps by uncovering under-researched pathogens, species, pathways, and regions, while highlighting challenges and data limitations in current assessments. Our review mapped the current landscape of QRA for disease introduction comparing trends and challenges across reviewed studies to promote QRA is a valuable decision-support tool and encourage the adoption of more diverse approaches. Furthermore, this study reveals opportunities for international collaboration and highlights innovative tools and models that can be adopted or refined.

## 5 Conclusion

This review of QRA literature, with focus on disease introduction, highlighted several gaps. There is a notable underrepresentation of certain diseases and countries, with underrepresentation of wildlife and aquatic animals, which creates gaps in global risk understanding and management. Additionally, many QRAs lack comprehensive consequence assessments, failing to fully capture the economic, social, and environmental impacts of potential risks. Most QRAs are focused on import risk assessments, potentially overlooking other critical areas and methodologies. By addressing the identified limitations and challenges, subsequent studies can build on the findings from this review to further improve and broaden the scope of QRA in animal health.

## Data Availability

The datasets presented in this study can be found in online repositories. The names of the repository/repositories and accession number(s) can be found in the article/Supplementary material.
