# Supplementary material for: Quantitative risk assessment for infectious disease introduction in animal populations: a comprehensive review
Source: Front Vet Sci. 2025 Oct 10;12:1648695. doi: 10.3389/fvets.2025.1648695 (PMC12551396; doi:10.3389/fvets.2025.1648695)
Supplement: Supplementary file 2 [file Supplementary_file_1.docx]

**Supplementary Material 1**

1. Import risk analysis

The QRAs that considered imports of companion animals, live animals and/or animal products were classified as IRAs, where IRA was classified separately when applied to the introduction through imported vectors or associated with the risk of exports. Publications on IRA totaled n=21, n=14 of which referring to the movement of livestock animals and/or their products, n=4 for companion animals and n=3 for poultry, here considered separately to the traditional definition of livestock. Furthermore, the final risk estimates were frequently annual, with n=4 providing both annual and monthly risks and only one IRA providing a monthly estimate. Most publication’s risk outputs were calculated at the country level, while n=5 publications provided estimates for multiple countries.

The outputs of the QRA’s varied, with n=6 providing not only an estimate for introduction but also for exposure, while n=2 for consequence. For these publications, only one provided economic losses and considered the effect of additional testing and culling of the infected animals (de Vos CJ *et al.*, 2015) while the other estimated only losses in terms of number of herds infected through import (Santman-Berends *et al.*, 2017). Additionally, neither of these IRAs included risk of exposure. The publications that accounted for the risk of exposure as well as introduction, provided outcome in several forms. For the one QRA that included exposure assessment for livestock products, the probability of introducing ASF into the country was expressed as a binomial process considering the probability of entry, the probability of exposure and the number of kilograms of pork and pork products on a monthly basis (Muñoz-Pérez C *et al.*, 2023). On the other hand, n=3 of the publications included the exposure in the assessment of probability of introduction: Mur et al (2012) determined that the probability of having at least one outbreak of ASF, included the probability of introducing an infected pig and having an effective contact from an infected to a susceptible pig at the destination; according to Miller et al (2012), the final probability of an outbreak due to importation included the estimate for the probability of establishing an effective contact capable of transmitting FMDV; similarly, Saegerman et al (2019) included the probability of transmission in the estimate for the annual probability of a first outbreak of LSD in France due to the legal transport of live cattle.

Most publications for QRA assessed only the probability of introduction, without reference to transmission and exposure (n=13). A common output was the number of years until the introduction of one infected animal or the probability of introducing at least one infected animal. When applied to companion animals, all n=4 publications made use of stochastic scenario-tree modeling and pathway scenarios in their QRA. Risk assessment for poultry made use of stochastic multilevel binomial risk models for estimating probability of introduction, accounting for uncertainty and variability of input. Multilevel binomial models, which account for more than one hierarchical level of animal aggregation, such as individual animals, herds, and regions, are best at capturing the complexity of disease dynamics across different scales of population structure, providing a more detailed and realistic understanding of how diseases spread but require quite granular information characterizing the animal populations. One paper in particular confronted the results from a multilevel binomial process considering herd-level effects and a one-level binomial process and a third ordinal model ranking countries in terms of the risk that they impose for of disease via the legal trade of live animals (Sánchez-Vizcaíno *et al.*, 2012), which performed with similar results though the ordinal model may be favored in the event of unavailable information for model parameterization.

To evaluate the effect of varying different input parameters, sensitivity analysis was performed for all but 1 publication which was a summary paper of a more detailed analysis (EFSA Panel on Animal Health and Welfare (AHAW), 2013). On the other hand, scenario analysis can be used to explore different potential outcomes based on varying assumptions and conditions. For IRA publications, we found intervention scenarios were performed for n=9 publications for increased detection probability, testing or importing from countries with varying prevalence, varying transmission rates, variation in parameters to assess reduced compliance, increased imports, increased cases in exporting country, vaccine efficacy in exporting country, policy, waiting period (exporting country) and incubation period.

1. Vectors introduced through imports

In assessing the risk of introducing vector-borne diseases through the importation of live animals and/or vectors, different approaches were employed. From the n=5 publications referring to vector importation as a pathway for introduction, three considered probability of introducing infected mosquito via truck, aircraft and/or cargo ships, one of which produced risk maps for exposure as vector host ratios in the UK for targeted surveillance and another producing (Brown *et al.*, 2012). Spatiotemporal modelling was also used to determine the mean probability of disease introduction via an infectious host or vector using a stochastic spatiotemporal model (Faverjon *et al.*, 2017). Regarding scenario analysis, n=4 publications explored increased prevalence in vectors, changes in quarantine period, testing, vector protection and importing restrictions, and changes in biosecurity practices such as on-site cleaning and disinsectisation, disinfection of trucks unloading of animals during long transportation periods, and probability of transmission at destination.

1. Exported animals or animal products

Most publications where risk was assessed for an exported live animal or animal product produced estimates of the probability of at least one infected animal or commodity being exported (De Vos *et al.*, 2010; Sergeant ES *et al.*, 2016; Meyer A *et al.*, 2017; Wang *et al.*, 2021; Zhang *et al.*, 2022). The exception was risk assessment for FMDV outbreaks in Russia (Wang *et al.*, 2021) which determined the probability of infection in the surveillance zone and the probability that at least one infected export per year from the surveillance zone. The model was self-assessed as semi-quantitative, but a quantitative estimate is provided for the probability of at least one FMD positive case exported from Russia and was thus included in the review. This model used the stochastic decision tree approach much like two other publications in this group. When it came to exploring variations in scenarios, n=3 publications considered variations in vaccination and screening, prevalence at origin and strategies for testing pre-export or post arrival.

1. Others

To assess the risk of disease introduction outside the scope of importations, imported vectors and exports, three publications were identified. One such publication is a spatial model using spatial multicriteria decision analysis (MCDA) to produce risk maps for High pathogenicity avian influenza (HPAI) H5N1 in domestic poultry where a suitability index ranging from 0 (least suitable) to 1 (highly suitable) is interpreted as a risk-like measure of the suitability for HPAI H5N1 occurrence (Paul *et al.*, 2016). Here, the final suitability maps were produced as a weighted linear sum applied to risk factor data layers (Paul *et al.*, 2016). A second publication in this category focused on between area movement of animals and determined the probability of entry of AHSV into a controlled area (Grewar *et al.*, 2021). Lastly, a QRA not related to imports, exports or imported vectors assessed the risk of infection at the wild and domestic animal interface as an annual probability of at least one bovine becoming infected with FMDV at the wildlife-livestock interface (Jori and Etter, 2016). This estimate incorporated the probability of contact between wild and domestic animals.

References

Brown, E.B. *et al.* (2012) ‘Assessing the risks of West Nile virus-infected mosquitoes from transatlantic aircraft: implications for disease emergence in the United Kingdom.’, *Vector borne and zoonotic diseases (Larchmont, N.Y.)*, 12(4), pp. 310–20. Available at: https://doi.org/10.1089/vbz.2010.0176.

De Vos, C.J. *et al.* (2010) ‘Probability of Exporting Infected Carcasses from Vaccinated Pigs Following a Foot‐and‐Mouth Disease Epidemic’, *Risk Analysis*, 30(4), pp. 605–618. Available at: https://doi.org/10.1111/j.1539-6924.2009.01327.x.

De Vos CJ *et al.* (2015) ‘Risk-based testing of imported animals: A case study for bovine tuberculosis in The Netherlands.’, *Preventive veterinary medicine*, 121(1), pp. 8–20. Available at: https://doi.org/10.1016/j.prevetmed.2015.04.017.

EFSA Panel on Animal Health and Welfare (AHAW) (2013) ‘Scientific Opinion on Rift Valley fever’, *EFSA Journal*, 11(4). Available at: https://doi.org/10.2903/j.efsa.2013.3180.

Faverjon, C. *et al.* (2017) ‘Comparative Risk Analysis of Two Culicoides-Borne Diseases in Horses: Equine Encephalosis More Likely to Enter France than African Horse Sickness.’, *Transboundary and emerging diseases*, 64(6), pp. 1825–1836. Available at: https://doi.org/10.1111/tbed.12577.

Grewar, J.D. *et al.* (2021) ‘An entry risk assessment of African horse sickness virus into the controlled area of South Africa through the legal movement of Equids’, *PLoS ONE*, 16(5). Available at: https://doi.org/10.1371/journal.pone.0252117.

Hall, R.N. *et al.* (2023) ‘A quantitative risk assessment for the incursion of lumpy skin disease virus into Australia via long-distance windborne dispersal of arthropod vectors’, *Preventive Veterinary Medicine*, 218, p. 105990. Available at: https://doi.org/10.1016/j.prevetmed.2023.105990.

Jori, F. and Etter, E. (2016) ‘Transmission of foot and mouth disease at the wildlife/livestock interface of the Kruger National Park, South Africa: Can the risk be mitigated?’, *Preventive Veterinary Medicine*, 126, pp. 19–29. Available at: https://doi.org/10.1016/j.prevetmed.2016.01.016.

Meyer A *et al.* (2017) ‘Movement and contact patterns of long-distance free-grazing ducks and avian influenza persistence in Vietnam.’, *PloS one*, 12(6), p. e0178241. Available at: https://doi.org/10.1371/journal.pone.0178241.

Miller, G.Y. *et al.* (2012) ‘Probability of introducing foot and mouth disease into the United States via live animal importation: -EN- -FR- Probabilité d’introduction de la fièvre aphteuse aux États-Unis suite à l’importation d’animaux vivants -ES- Probabilidad de introducción de la fiebre aftosa en los Estados Unidos por la importación de animales vivos’, *Revue Scientifique et Technique de l’OIE*, 31(3), pp. 777–787. Available at: https://doi.org/10.20506/rst.31.3.2154.

Muñoz-Pérez C *et al.* (2023) ‘Quantitative risk assessment of African swine fever introduction into Spain by legal import of swine products.’, *Research in veterinary science*, 163, p. 104990. Available at: https://doi.org/10.1016/j.rvsc.2023.104990.

Mur, L. *et al.* (2012) ‘Quantitative Risk Assessment for the Introduction of African Swine Fever Virus into the European Union by Legal Import of Live Pigs: Import Risk Assessment for ASFV into EU’, *Transboundary and Emerging Diseases*, 59(2), pp. 134–144. Available at: https://doi.org/10.1111/j.1865-1682.2011.01253.x.

Paul, M.C. *et al.* (2016) ‘Quantitative assessment of a spatial multicriteria model for highly pathogenic avian influenza H5N1 in Thailand, and application in Cambodia’, *Scientific Reports*, 6(1), p. 31096. Available at: https://doi.org/10.1038/srep31096.

Saegerman, C. *et al.* (2018) ‘Risk of introduction of lumpy skin disease in France by the import of vectors in animal trucks’, *PLoS ONE*, 13(6). Available at: https://doi.org/10.1371/journal.pone.0198506.

Saegerman C *et al.* (2019) ‘Risk of introduction of Lumpy Skin Disease into France through imports of cattle.’, *Transboundary and emerging diseases*, 66(2), pp. 957–967. Available at: https://doi.org/10.1111/tbed.13111.

Sánchez-Vizcaíno, F. *et al.* (2012) ‘Comparative Assessment of Analytical Approaches to Quantify the Risk for Introduction of Rare Animal Diseases: The Example of Avian Influenza in Spain’, *Risk Analysis*, 32(8), pp. 1433–1440. Available at: https://doi.org/10.1111/j.1539-6924.2011.01744.x.

Santman-Berends, I.M.G.A. *et al.* (2017) ‘A quantitative risk-analysis for introduction of Bovine Viral Diarrhoea Virus in the Netherlands through cattle imports’, *Preventive Veterinary Medicine*, 146, pp. 103–113. Available at: https://doi.org/10.1016/j.prevetmed.2017.08.003.

Sergeant ES *et al.* (2016) ‘Quantitative Risk Assessment for African Horse Sickness in Live Horses Exported from South Africa.’, *PloS one*, 11(3), p. e0151757. Available at: https://doi.org/10.1371/journal.pone.0151757.

Taylor, R.A. *et al.* (2019) ‘A generic framework for spatial quantitative risk assessments of infectious diseases: Lumpy skin disease case study’, *Transboundary and Emerging Diseases*, 66(1), pp. 131–143. Available at: https://doi.org/10.1111/tbed.12993.

Wang, J. *et al.* (2021) ‘Risk assessment and integrated surveillance of foot-and-mouth disease outbreaks in Russia based on Monte Carlo simulation’, *BMC Veterinary Research*, 17(1), p. 268. Available at: https://doi.org/10.1186/s12917-021-02967-x.

Zhang, S. *et al.* (2022) ‘Epidemiologic and import risk analysis of Peste des petits ruminants between 2010 and 2018 in India’, *BMC Veterinary Research*, 18(1), p. 419. Available at: https://doi.org/10.1186/s12917-022-03507-x.
